# Implications for human adipose-derived stem cells in plastic surgery

**DOI:** 10.1111/jcmm.12425

**Published:** 2014-11-26

**Authors:** Derek A Banyard, Ara A Salibian, Alan D Widgerow, Gregory R D Evans

**Affiliations:** a Department of Plastic Surgery, University of California, IrvineOrange, CA, USA

**Keywords:** stem cell, mesenchymal stem cell, adipose-derived stem cell, lipotransfer

## Abstract

Adipose-derived stem cells (ADSCs) are a subset of mesenchymal stem cells (MSCs) that possess many of the same regenerative properties as other MSCs. However, the ubiquitous presence of ADSCs and their ease of access in human tissue have led to a burgeoning field of research. The plastic surgeon is uniquely positioned to harness this technology because of the relative frequency in which they perform procedures such as liposuction and autologous fat grafting. This review examines the current landscape of ADSC isolation and identification, summarizes the current applications of ADSCs in the field of plastic surgery, discusses the risks associated with their use, current barriers to universal clinical translatability, and surveys the latest research which may help to overcome these obstacles.

- Introduction
- Methods of ADSC isolation & identification
- Current applications
  – Soft tissue
  – Bone and cartilage
  – Wound healing
  – Peripheral nerve regeneration
- Barriers to clinical entry
- Future directions
- Concluding remarks

## Introduction

Recent advances in regenerative medicine, in particular the discovery of multipotent, easily accessible stem cells such as adipose-derived stem cells (ADSCs), have provided the opportunity of using autologous stem cell transplants as regenerative therapies. The field of plastic surgery, centred on the restoration and enhancement of the body, is logically positioned to utilize such new technologies focused on the repair and replacement of diseased cells and tissues [1]. The ability of stem cells to self-renew, to secrete trophic factors and to differentiate into different cell types has allowed for the development of more flexible therapies to redefine the classic ‘autologous tissue transplant’ and offer more customizable treatment options. ADSCs are being utilized for a variety of different applications in plastic surgery [2–11], and as our understanding of the basic science of stem cells continues to develop, the plastic surgeon should be prepared for the translational and clinical implications of this progress.

Adipose-derived stem cells are particularly useful as they can be easily harvested with minimal donor site morbidity and have a differentiation potential similar to other MSCs [12,13]. In addition, ADSCs have higher yields and greater proliferative rates in culture when compared to bone marrow stromal cells [14–16]. The discovery that ADSCs are not only precursors to adipocytes but also are multipotent progenitors to a variety of cells [17] including osteoblasts, chondrocytes, myocytes, epithelial cells and neuronal cells [18], creates the potential to treat a variety of tissue defects from a single, easily accessible autologous cell source.

Adult stem cell research has made significant strides as a therapeutic modality in recent years. However, there remain significant barriers to the safe and efficacious use of stem cell therapies. With regard to ADSCs, this includes better defining the source population of multipotent cells, optimizing the isolation of these cells in compliance with regulatory standards, and better understanding the behaviour of ADSCs in their transplanted niche. The purpose of this review is to (*i*) explore the utilization of ADSCs in plastic surgery, (*ii*) describe the current limitations of ADSC treatments with regard to developing translatable clinical therapies and (*iii*) describe certain techniques used in our laboratory that may help overcome these barriers. Understanding the current status of clinical ADSC treatments and defining the challenges ahead may bring us closer to achieving desired outcome while minimizing unwanted side effects with these therapies.

## Methods of ADSC isolation & identification

The most commonly published method of ADSC isolation involves enzymatic digestion of lipoaspirate to release the stromal vascular fraction (SVF) of cells which include stromal & endothelial cells, pericytes, various white blood cells, red blood cells and stem/progenitor cells [19]. The enzyme preparations used to achieve this fraction include dispase, trypsin and more commonly collagenase. In our laboratory, we take freshly harvested lipoaspirate and wash it with sterile 1% PBS until golden in colour. The adipose tissue is then digested with 0.01% collagenase/PBS solution at a ratio of 1 ml of enzyme solution to 1 cm^3^ of adipose tissue. This mixture is incubated at 37°C with intermittent agitation until it becomes cloudy (usually 30 min.). The infranatant is then carefully aspirated, transferred to 50 ml conical tubes and centrifuged at 706 × g for 8 min. The supernatant is discarded and resulting pellet, the SVF, is resuspended in control media [DMEM supplemented with 10% foetal bovine serum (FBS), 500 IU penicillin and 500 μg streptomycin; Mediatech, Manassas, VA, USA]. The cells are then counted and plated in uncoated T75 flasks at a concentration of 1 × 10^6^ cells. Consistently, 20 mg of lipoaspirate is ample tissue to harvest an adequate yield of SVF (>1 × 10^7^ cells).

In 2006, the International Society for Cellular Therapy (ICTS) defined a set of minimal criteria for identifying cells as ADSCs. These include plastic adherence while maintained in standard culture conditions, expression of CD73, CD90 and CD105 while lacking the expression of CD45, CD34, CD14 or CD11b, CD79α or CD19 and HLA-DR surface molecules [20]. In conjunction with the International Federation for Adipose Therapeutics and Science in 2013, the ICTS has denoted additional surface markers CD13, CD29 and CD44 as being constitutively expressed at >80% on the surface of ADSCs, while CD31, CD45 and CD235a are the primary negative markers that should be expressed on less than 2% of the cells [19]. Ultimately, the viability of the isolated cells should exceed 70% and the presence of at least two positive and two negative markers are necessary for foundational phenotyping. Finally, ADSCs must possess the ability to differentiate into osteoblasts, adipocytes and chondroblasts.

**Fig. 1:**
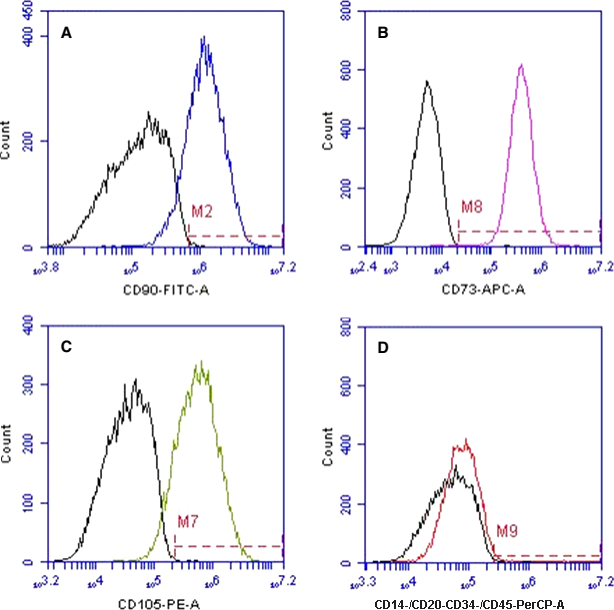
Flow cytometry analysis of isolated ADSCs after collagenase method. Cells stained (**A**) 81.3% positive for CD90, (**B**) 99.9% positive for CD73, (**C**) 86.6% positive for CD105 and (**D**) 1.97% positive for CD14, CD20, CD34 and CD45.

**Fig. 2:**
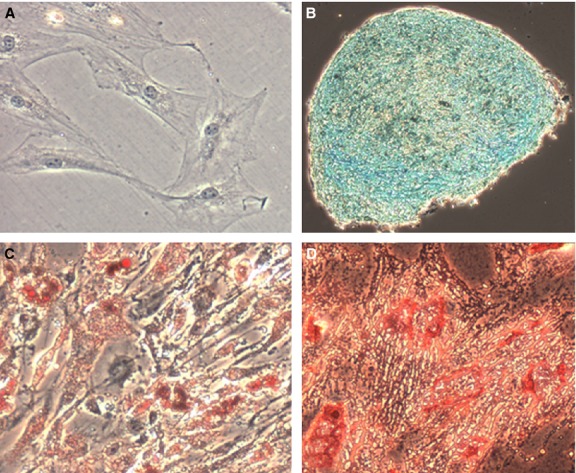
Undifferentated and differentiated ADSCs visualized using microscopy. Original magnification, 10×. (**A**) Control stain – uADSCs stained with Oil Red O (other controls not shown). (**B**) Staining with Alcian Blue revealing presence of chondroblasts. (**C**) Staining with Oil Red O revealing presence of adipocytes. (**D**) Staining with Alizarin Red S revealing presence of osteoblasts.

Identification of ADSCs in our laboratory is accomplished by labelling our plastic-adherent cells with a mesenchymal stem cell (MSC) phenotyping kit after the second passage (Miltenyi Biotec Inc, Auburn, CA, USA). Cells are analysed using a C6 Accuri Flow Cytometer (BD Biosciences, San Jose, CA, USA) which demonstrate positive staining for CD90 (81.3%), CD105 (86.6%) and CD73 (99.9%) and negative staining for CD14, CD20, CD34 and CD45 (1.97% – Fig. 1). To complete the identification of our ADSCs, we culture these cells in adipogenic, osteogenic, or chondrogenic conditions provided in commercially available kits (Cyagen Biosciences Inc., Sunnyvale, CA, USA). Cells subjected to adipogenic or osteogenic conditions reveal lipid droplets or calcium synthesis after staining with Oil Red O or Alizarin Red S, respectively, after fixation in 4% formalin. Cells subjected to chondrogenic conditions reveal proteoglycan synthesis upon staining with Alcian Blue after paraffin embedding (Fig. 2). The ease at which ADSCs can be isolated has led to rapid and widespread translational applications.

A number of groups have described the isolation of ADSCs using non-enzymatic methods. Studies show that ADSCs reside in the infranatant layer of the suction canister after liposuction and that these cells can be expanded *ex vivo*. And while these cells exhibit phenotypic and differentiation potential similar to ADSCs isolated *via* collagenase digestion, their presence is significantly lower with reported yields ranging from a 3- to 19-fold decrease in comparison [21–24]. Interestingly, it has been found that multiple variables, including medical comorbidities of the patient, location adipose tissue stores, and the method in which this tissue is harvested, all affect the properties of the ADSCs therein. For example, diabetic patient fat stores have been found to contain fewer ADSCs with a reduced phenotypic expression profile and ability to proliferate [3]. The anatomical location of adipose tissue harvest also appears to have an effect on the yield and characteristics of the isolated ADSCs [25,26]. More recently, Gnanasegaran *et al*. demonstrated that the gene expression levels and tendency towards specific germ layer differentiation is affected by whether the fat is harvested *via* liposuction *versus* lipectomy [27].

## Clinical applications

### Soft tissue

The regenerative potential of ADSCs has been demonstrated for several years with techniques such as fat grafting for replacement of soft tissue. The autologous tissue utilized in fat grafting contains a variety of cells, including ADSCs [28], which support tissue neo-vascularization [29] and regeneration through secretion of angiogenic growth factors [30]. Cell-assisted lipotransfer (CAL), a technique first described by Matsumoto *et al*. in 2006, combines aspirated fat with concentrated ADSCs to create stem cell-rich fat grafts [5]. This approach allows for marked improvements in the survival rate of transplanted fat with less fat resorption [31] as well as a decrease in adverse effects of lipoinjection such as fibrosis and cyst formation [5]. In breast tissue, fat grafting has also been associated with fat necrosis and calcifications which can complicate breast imaging and breast cancer surveillance [32,33]. Coincidentally, Yoshimura *et al*. used CAL in 2008 for cosmetic breast augmentation in 40 patients and reported favourable aesthetic outcome with no major complications [10]. Since then, CAL has been used in other studies for breast augmentation [2,34,35] as well as for facial lipoatrophy [11,36] and augmentation during face-lift and facial contouring surgeries, among other applications (Table 1) [37].

**Table 1:** Clinical applications of ADSCs

Application	Source articles
Soft tissue reconstruction	
Breast augmentation	Kamakura *et al*., 2011*; Tiryaki *et al*., 2011†; Wang *et al*., 2012*; Yoshimura *et al*., 2008*
Breast augmentation revision	Yoshimura *et al*., 2010*
Facial lipoatrophy	Castro-Govea *et al*., 2012*; Tiryaki *et al*., 2011†; Yoshimura *et al*., 2008*
Facial augmentation	Lee S. *et al*., 2012
Lumpectomy reconstruction	Tiryaki *et al*., 2011†
Traumatic/iatrogenic soft tissue defects	Tiryaki *et al*., 2011†
Bony reconstruction	
Calvarial reconstruction	Lendeckel *et al*., 2004; Thesleff *et al*., 2011
Maxilla reconstruction	Mesimaki *et al*., 2009
Wound healing	
Radiation atrophy	Rigotti *et al*., 2007
Ischaemic wounds	Lee H. *et al*., 2012

* Cell-assisted lipotransfer (CAL).

† Stem cell-enriched tissue injections.

ADSC, Adipose-derived stem cell.

Most recently, Kolle *et al*. demonstrated significant fat graft survival in human cases after *ex vivo* expansion of collagenase-processed ADSCs used for CAL when compared to the traditional method of fat grafting [38]. Alternative ADSC therapies have also been explored including techniques such as stem cell-enriched tissue injections that combine traditional fat grafts and subsequent ADSC injections [39,40]. These preliminary studies suggest that ADSCs might allow for improvements in the retention and volume-restoring capabilities of transplanted fat, though the applicability of these studies in the United States is limited because of the methods (enzymatic isolation) used.

Concern has been raised over the use of ADSCs in the form of CAL for breast augmentation after breast cancer therapy. For example, studies have demonstrated that MSCs potentiate the metastatic potency of breast cancer cells when the two are mixed and reimplanted [41–43]. While there is little evidence with regard to CAL therapy for breast augmentation post-mastectomy, the American Society of Plastic Surgeons has come to the conclusion that fat grafting does not increase the risk of breast cancer recurrence [44]. It appears that ADSCs may enhance the growth of active cells without affecting dormant cells, but because there is a need for more research in this setting, the current recommendations are such that CAL therapy should be delayed for reconstructive purposes in breast cancer treatment until there is no evidence of active disease for a period of 7 years [45].

### Bone and cartilage

Mesenchymal stem cells were first identified in the bone marrow (BM-MSCs), but the costs of harvesting these cells seemed to rarely outweigh the benefits of their use. The discovery of MSCs in adipose tissue was met with great excitement. ADSCs harvest is safer, easier and yields as high as 500 times more cells than when harvesting stem cells from the same amount of BM tissue [46]. And while ADSCs are known to exhibit some differences in phenotypic, transcriptome and proteome expression when compared to BM-MSCs (*e.g*. CD34 expression), ADSCs have been found to have superior differentiation, proliferation and immunomodulatory effects [46].

Adipose-derived stem cells, by definition, are able to differentiate into osteoblasts and chondroblasts and have therefore been explored for bone and cartilage regeneration therapies. Current clinical stem cell therapies for bone regeneration have demonstrated promising results for craniofacial defects [4,8,47]. Studies have shown that ADSCs, either combined with autologous bone [4] or seeded alone in β-tricalcium phosphate (TCP) granules [8] are capable of forming new bone and repairing large calvarial defects in human cases. Stem cell treatments have also been used for repair of defects involving the maxilla and mandible. Multistep delayed procedures that combine ADSCs with growth factors [47] in muscle tissue, followed by transplanting the entire structure as a composite microvascular flap surrounding ectopic bone have yielded excellent functional and aesthetic results in maxilla repair [47]. Single-stage procedures involving ADSCs seeded on scaffolds of β-TCP and bone morphogenetic protein 2 have also been used to fill a mandibular defect [48]. Overall, these studies suggest that ADSCs are capable of ossifying bony defects and providing a non-invasive method of bony reconstruction without the associated donor site morbidity of traditional bone grafts [49].

Cartilage defects present a challenging reconstructive problem because of the tissue's limited intrinsic capacity for self-repair. To the best of the authors’ knowledge, there are no clinical trials utilizing ADSCs to treat cartilage defects, though several animal studies have yielded promising results. ADSCs cultured in a three-dimensional environment [50] and preconditioned with the appropriate growth factors, primarily those in the TGF-β superfamily [51], are capable of forming cartilage tissue *in vivo* [52]. In addition, undifferentiated ADSCs have fully repaired hyaline cartilage defects in patellofemoral joints [53] and ear auricle defects [54] in animals. The latter experiments suggest the intrinsic ability of ADSCs to adapt to their environment *in vivo* and create a promising direction for future clinical applications.

### Wound healing

Adipose-derived stem cells are favourable candidates for wound therapies as they secrete numerous growth factors and cytokines critical in wound healing [55,56] and also increase macrophage recruitment, enhance granulation tissue, and improve vascularization (Table 2) [57,58]. Repeated transplants of purified autologous lipoaspirates into radiation-induced lesions in breast cancer patients have shown improvement of ultrastructural tissue characteristics with neovessel formation as well as significant clinical improvements [6]. ADSCs have also been used clinically to treat wounds complicated by ischaemia, such as in thromboangiitis obliterans and diabetes [3]. The angiogenic properties of ADSCs may contribute to the collateral vessel formation seen in these patients.

**Table 2:** Selected ADSC secretomes and their functions

Growth factor	Function	Source articles
Brain-derived growth factor (BDNF)	Nerve regeneration	Salgado *et al*., 2010; Lopatina *et al*. 2011; Reid *et al*., 2011; Sowa *et al*., 2012; Kingham *et al*., 2013
Glial-derived growth factor (GDNF)	Nerve regeneration	Salgado *et al*., 2010; Reid *et al*., 2011; Lopatina *et al*. 2011; Kingham *et al*., 2013
Hepatocyte growth factor (HGF)	Angiogenesis, wound healing, immunomodulation	Wang *et al*., 2006; Kapur *et al*., 2013
Insulin-like growth factor-1 (IGF-1)	Wound healing, nerve regeneration, cardiac regeneration	Wang *et al*., 2006; Salgado *et al*., 2010; Kapur *et al*., 2013
Nerve growth factor (NGF)	Nerve regeneration	Salgado *et al*., 2010; Reid *et al*., 2011; Lopatina *et al*. 2011; Sowa *et al*., 2012
Vascular endothelial growth factor (VEGF)	Angiogenesis, wound healing, cardiac regeneration, immunomodulation	Wang *et al*., 2006; Sowa *et al*., 2012; Kingham *et al*., 2013; Kapur *et al*.,2013
Transforming Growth Factor beta (TGF-β)	Angiogenesis, immunomodulation	Salgado *et al*., 2010; Lopatina *et al*. 2011; Kapur *et al*., 2013
Basic Fibroblast Growth Factor (bFGF)	Angiogenesis	Salgado *et al*., 2010; Kapur *et al*., 2013
Granulocyte colony-stimulating factor (G-CSF)	Angiogenesis, wound healing Wound healing	Kapur *et al*., 2013
Interleukin 6 (IL-6)	Immunomodulation	Kapur *et al*., 2013
Interleukin 8 (IL-8)	Wound healing	Kapur *et al*., 2013

ADSC, Adipose-derived stem cell.

In addition, ADSCs have also shown to be useful in treating pathological wound healing such as aberrant scar formation. Scars treated with ADSC injections in animal models exhibited subsequent reduction in surface area and improvements in colour and pliability when compared to controls [59]. Potential mechanisms of decreased scarring may involve targeting of the inflammatory processes associated with scar formation [60] as ADSCs have been shown to have anti-inflammatory and immunosuppressive effects [61,62].

### Peripheral nerve regeneration

The use of autologous nerve grafts for the repair of peripheral nerve injuries (PNI) is limited by donor site morbidity and suboptimal functional recovery. As a result, alternative treatments have been investigated including several forms of regenerative and cellular therapies. The majority of research on PNI has focused on replacing host support cells, particularly the Schwann-cell (SC) population, as these cells are crucial in providing trophic, structural and directional support for regenerating axons [13]. In addition, *in vivo* studies have demonstrated that ADSCs can promote nerve regeneration by differentiating into neuron-like lineages [7].

Recent PNI research has shifted focus to the role of ADSCs providing support to host cells. In this setting, *in vivo* and *in vitro* studies that include the transplantation of undifferentiated ADSCs (uADSCs), and even ADSC-conditioned media, have demonstrated mechanisms of neurotrophic factor elaboration, including glial-derived growth factor, nerve growth factor, brain-derived growth factor, glial cell-derived neurotrophic factor, insulin-like growth factor, hepatocyte growth factor and VEGF as promoting significant nerve regeneration [63–67]. These findings support a role for ADSCs in providing a favourable microenvironment to support regenerating axons *via* paracrine mechanisms. In our laboratory, preliminary data support this paracrine role and indicates that undifferentiated ADSCs can be modulated towards neurotrophic secretome function when cultured in embryonic motor neuron-conditioned media. The current trends in PNI research with ADSCs and demonstration of nerve regeneration in many *in vivo* models indicate that clinical trials may be on the horizon.

## Barriers to clinical entry

In Europe, ADSCs are considered Advanced Therapy Medicinal Products, as defined by the European Union (European Commission) 1394/2007 which contains rules for ‘authorization, supervision, and pharmacovigilance’ regarding the summary of product characteristics, labelling, and packaging of Advanced Therapy Medicinal Products that are prepared commercially and in academic institutions [68]. This regulation refers to the European good manufacturing process (eGMP) rules [69]. The process of converting protocols, including collagenase-processed ADSCs, into a process that is compliant with eGMP requires assays that have had careful consideration of all the risks and benefits for the patient end user. As a result, the general recommendation on the use of enzyme-processed CAL in the clinical setting is not prohibited as this technique has been demonstrated to provide satisfying results in terms of long-term outcome, most likely because of the dramatic release of angiogenic growth factors and the differentiation of ADSCs into adipocytes and vascular endothelial cells [5,10,11].

In the United States, the Food and Drug Administration (FDA) regulates Human Cells and Tissue-Based Products (HCT/P) intended for human transplant and maintains two levels of classifications: 361 and 351 products. HCT/P 361 encompasses ‘tissue’ (*e.g*. bone, ligaments, vein grafts, *etc*.) and their related procedures that take place in the same operative session, all of which fall under the jurisdiction of practice of medicine which is governed by state medical boards and professional societies; not the FDA. HCT/P 351, on the other hand, includes ‘drugs/biologics’ (*e.g*. cultured cells, lymphocyte immune therapy, cell therapy involving the transfer of genetic material, *etc*.) which is fully governed by FDA [70,71]. Regulation 21 CFR 1271 directly demonstrates the FDA's position on enzymatically isolated adipose stem cells derived from SVF for reconstructive purposes as beyond the scope of ‘minimal manipulation’ and therefore, a drug [72]. Thus, the practical implication is the need for any surgeon who wishes to use ADSCs isolated *via* collagenase to submit an Investigational New Drug application to the FDA and have an approved Institutional Review Board with the referring Institution.

Given the time, expense and complexity of the regulatory issues surrounding ADSCs intended for transplantation, it is evident that U.S. physicians are discouraged to perform any cell-supplemented lipotransfer techniques in the current commonly accepted practices. Furthermore, automated devices for separating adipose stem cells are regulated as class III medical devices by the FDA, and currently, none are approved for human use in the United States. Kolle *et al*. demonstrated that CAL, when supplemented with ADSCs expanded *ex vivo* after collagenase digestion, yields superior results when compared to lipotransfer alone [38]. The FDA restrictions that would preclude such a study to be conducted in the United States prompt an impetus to develop methods for CAL that results in ‘minimal manipulation’ of source adipose tissue.

## Future directions

**Fig. 3:**
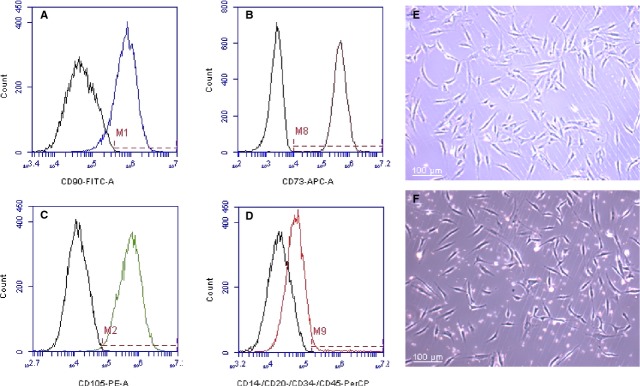
Flow cytometry analysis of isolated ADSCs after rapid isolation (no collagenase). Cells stained (**A**) 85.8% positive for CD90, (**B**) 99.9% positive for CD73, (**C**) 99.4% positive for CD105 and (**D**) 3.79% positive for CD14, CD20, CD34 and CD45. (**E**) Collagenase-isolated ADSCs after 6 days of primary culture seeded at 5 × 10^5^ in T75 flask. (**F**) Rapid isolation ADSCs after 6 days primary culture seeded at 2 × 10^6^ in T75 flask.

In 2006, Yoshimura *et al*. described a cell population in the liposuction aspirate fluid that exhibited similar phenotypic properties to ADSCs harvested in the traditional manner (collagenase) from processed lipoaspirate cells; however, the yield was reduced by a third when comparing to the two methods [23]. Since that time, additional studies have been published touting the benefits of non-enzymatic ADSC isolation. In 2010, Francis *et al*. described a method of ADSC ‘Rapid Isolation’ in ∼30 min. that excluded the use of collagenase, however, a significant disadvantage of this study was the low yield of ∼250,000 cells from a starting volume of ∼250 ml liposuction aspirate fluid [21]. Zeng *et al*. describe a ‘rapid and efficient’ form of non-enzymatic ADSC isolation in which adipose tissue is cut into tiny pieces and placed in culture flasks with 100% FBS in which the plastic-adherent cells were allowed to expand over a period of days [24]. One obvious downside to this method is the requirement to expand the cell population in calf serum. Most recently, Shah *et al*. describe a form of non-enzymatic ADSC isolation combining the cells of the liposuction aspirate fluid with the cells captured from the processed lipoaspirate tissue wash that is typically discarded prior to collagenase digestion [22]. They observed significant improvement in MSC-related phenotypic markers and similar adipogenic and osteogenic differentiation characteristics. While their isolation time was cut by one-third, they observed a 19-fold decrease in ADSC isolation when compared to the traditional method. In our laboratory, we have adopted a very similar protocol of non-enzymatic isolation that includes processing the processed lipoaspirate effluent. The primary difference in our protocol, however, is the method of plating cells. While Shah *et al*. plate the entire SVF pellets in T175 flasks, we resuspend our pellets in culture media and then plate the cells at specific concentrations. In one experiment for example, we plated the SVF pellet after collagenase digestion at a concentration of 5 × 10^5^ in a T75 flask. Concurrently, we plated the SVF pellet obtained after non-enzymatic isolation at 2 × 10^6^. After 6 days of culture, these two flasks appeared nearly identical in terms of confluence, correlating to a fourfold decrease in ADSC harvest when using the latter method. The two cell populations were then analysed under flow cytometry as previously described. There is little difference in the phenotypic expression between the two populations as demonstrated by >80% expression of CD90, CD73 and CD105 and <5% expression of CD14, CD20, CD 34 and CD45 (Fig. 3).

Most convincingly, Kolle *et al*. demonstrated a clear benefit to CAL over lipotransfer alone. They isolated and expanded ADSCs *ex vivo* from human cases followed by lipotransfer to the cases’ arms with or without ADSC supplementation. They demonstrated a 65% improvement in fat graft survival after 4 months in the experimental group [38]. The major drawback to their experimental model was that to achieve these results, the 34 ml of lipotransfer was supplemented with 6.5 × 10^8^ ADSCs or 2000 times the physiological level [38]. The methods of ‘rapid isolation’, previously mentioned, demonstrate the ability to isolate ADSCs without the aid of enzymatic digestion, but at a cost of greatly reduced yields. There is significant doubt that ADSCs used at such low concentrations would serve for any clinical benefit. As previously discussed, *ex vivo* expansion of ADSCs is not practical for application in the United States or other principalities with strict regulations. Therein lies an impetus to discover innovative methods of ADSC isolation and characterization of the regenerative components of the SVF that might yield similar results to concentrated ADSCs alone.

There is promise in capitalizing on the plastic-adherent properties of ADSCs as a form of non-enzymatic isolation. The same group that first described the isolation of cells from the LAF, Doi *et al.,* has demonstrated that an adherent column of rayon–polyethylene non-woven fabrics may also be used to isolate ADSCs, though at an inferior yield to the traditional method [73]. Further advancements in harnessing the plastic-adherent properties of these cells are clearly needed as Buschmann *et al*. demonstrated that 30–50% of ADSCs remain in suspension after 24 hrs of primary culture [74].

## Concluding remarks

Many questions remain unanswered. A consensus on the phenotypic characterization of these ADSCs is still lacking as is a common method of isolation that will allow for direct translational applications worldwide. In addition, the long-term safety of CAL in areas of previous cancer remains unanswered. While we are still uncovering the exact mechanism of stem cell function, be it paracrine or differentiation induced, the discovery of the very abundant ADSC will allow for major advancements in regenerative medicine, particularly pertaining to therapeutics. To be truly translational, this research is challenged with producing minimally manipulated cells that can be used in the operating room either autogenously or in ‘off the shelf’ variations. In addition, it may well become clear that these therapies will need to be individually tailored if we discover that patients vary in their response to these cells or the trophic factors they produce. The ultimate answer will likely be achieved by the combined efforts of basic scientists, clinicians and biomedical engineers.
